# Sex differences in frontal lobe connectivity in adults with autism spectrum conditions

**DOI:** 10.1038/tp.2017.9

**Published:** 2017-04-11

**Authors:** E A Zeestraten, M C Gudbrandsen, E Daly, M T de Schotten, M Catani, F Dell'Acqua, M-C Lai, A N V Ruigrok, M V Lombardo, B Chakrabarti, S Baron-Cohen, C Ecker, MRC AIMS Consortium, Anthony J Bailey, Simon Baron-Cohen, Patrick F Bolton, Edward T Bullmore, Sarah Carrington, Marco Catani, Bhismadev Chakrabarti, Michael C Craig, Eileen M Daly, Sean C L Deoni, Christine Ecker, Francesca Happé, Julian Henty, Peter Jezzard, Patrick Johnston, Derek K Jones, Meng-Chuan Lai, Michael V Lombardo, Anya Madden, Diane Mullins, Clodagh M Murphy, Declan G M Murphy, Greg Pasco, Amber N V Ruigrok, Susan A Sadek, Debbie Spain, Rose Stewart, John Suckling, Sally J Wheelwright, Steven C Williams, C Ellie Wilson, D G M Murphy, M C Craig

**Affiliations:** 1 Department of Forensic and Neurodevelopmental Sciences, Institute of Psychiatry, Psychology and Neuroscience, King's College London, London, UK; 2 Autism Research Centre, Department of Psychiatry, University of Cambridge, Cambridge, UK; 3 Child and Youth Mental Health Collaborative at the Centre for Addiction and Mental Health and The Hospital for Sick Children, Department of Psychiatry, University of Toronto, Toronto, ON, Canada; 4 Department of Psychiatry, National Taiwan University Hospital and College of Medicine, Taipei, Taiwan; 5 Department of Psychology and Center for Applied Neuroscience, University of Cyprus, Nicosia, Cyprus; 6 School of Psychology and Clinical Language Sciences, Centre for Integrative Neuroscience and Neurodynamics, University of Reading, Reading, UK; 7 CLASS Clinic, Cambridgeshire and Peterborough NHS Foundation Trust, Cambridge, UK; 8 Sackler Institute for Translational Neurodevelopment, Institute of Psychiatry, King's College London, London, UK; 9 National Autism Unit, Bethlem Royal Hospital, SLAM NHS Foundation Trust, London, UK

## Abstract

Autism spectrum conditions (ASC) are more prevalent in males than females. The biological basis of this difference remains unclear. It has been postulated that one of the primary causes of ASC is a partial disconnection of the frontal lobe from higher-order association areas during development (that is, a frontal ‘disconnection syndrome'). Therefore, in the current study we investigated whether frontal connectivity differs between males and females with ASC. We recruited 98 adults with a confirmed high-functioning ASC diagnosis (61 males: aged 18–41 years; 37 females: aged 18–37 years) and 115 neurotypical controls (61 males: aged 18–45 years; 54 females: aged 18–52 years). Current ASC symptoms were evaluated using the Autism Diagnostic Observation Schedule (ADOS). Diffusion tensor imaging was performed and fractional anisotropy (FA) maps were created. Mean FA values were determined for five frontal fiber bundles and two non-frontal fiber tracts. Between-group differences in mean tract FA, as well as sex-by-diagnosis interactions were assessed. Additional analyses including ADOS scores informed us on the influence of current ASC symptom severity on frontal connectivity. We found that males with ASC had higher scores of current symptom severity than females, and had significantly lower mean FA values for all but one tract compared to controls. No differences were found between females with or without ASC. Significant sex-by-diagnosis effects were limited to the frontal tracts. Taking current ASC symptom severity scores into account did not alter the findings, although the observed power for these analyses varied. We suggest these findings of frontal connectivity abnormalities in males with ASC, but not in females with ASC, have the potential to inform us on some of the sex differences reported in the behavioral phenotype of ASC.

## Introduction

Autism spectrum conditions (ASC) affect ~1% of the UK population,^1^ with a male:female prevalence ratio estimated at 2–5:1.^2^ The cause(s) of this sex difference remains unclear.^2^ One putative explanation is that only the most ‘severe' or evident cases of females with ASC are diagnosed, as it is thought females may be more able to compensate for, or mask, their disabilities related to autism.^3, 4, 5, 6, 7^ Others have argued that ASC in females is not more severe, but represents a partially different behavioral phenotype,^7^ which may be under-detected by current diagnostic criteria.^8^ Demand avoidance and extreme determination are, for example, more commonly associated with the behavioral phenotype in females with ASC.^3, 4^ The limited neuroimaging studies to date, have further shown that in different age ranges, neuroanatomical features of ASC in females seem to involve different structures or growth trajectories than males with ASC.^9, 10, 11, 12, 13, 14, 15^ However, to date there have been insufficient well-powered studies into the neurological basis of sex differences in ASC. This has contributed to the current difficulties in our understanding for the roots of the skewed male:female prevalence ratio.

Previous structural neuroimaging studies in females with ASC^10, 16, 17^ reported little overlap of atypical brain areas found in meta-analyses of predominantly male samples.^18, 19^ Further, we recently reported significant differences in the regional gray and white matter neuroanatomy of ASC when directly studying differences between adult males and females with ASC.^10^ However, advances in neuroimaging technology have enabled research to focus on the brain as a network of connections. Also, it has been postulated that one of the primary causes of ASC is underpinned by a partial disconnection of the frontal lobe from higher-order association areas during development.^20, 21, 22^ Studies of connectivity in ASC, using for example diffusion tensor imaging (DTI) tractography to visualize connectivity fiber tracts are of great research interest.

The hypothesis that ASC is associated with a frontal disconnection syndrome has been supported by DTI tractography and tract-based spatial statistics (TBSS) studies. These studies have reported differences in the microstructure of tracts such as the inferior fronto-occipital fasciculus (IFOF) and uncinate fasciculus (UF) in ASC.^22, 23, 24^ White matter (WM) tracts central to language, the arcuate fasciculus (AF),^22^ and socioemotional processing, such as the inferior longitudinal fasciculus (ILF), have also been shown to have reduced FA in male-only or male-dominated studies of ASC.^23, 25, 26, 27^

However, previous studies often focused on males with ASC, and it remains unclear whether these differences also exist in females with ASC. In the light of our previous findings we hypothesized there would be minimal overlap in these tracts when analyzing how males and females with ASC, respectively, differ from typically developing males and females. If correct, this would lend support to the hypothesis that sex differences in behavioral phenotype in ASC are, in part, underpinned by differences in brain connectivity.

## Materials and methods

### Participants and assessment

Sixty-one right-handed male adults with a diagnosis of ASC (mean age: 26.0±7.0 years; range: 18–41), 61 neurotypical male controls (mean age: 28.5±6.8 years; range: 18–45), 37 adult ASC females (mean age: 25.4±6.1 years; range: 18–37) and 54 neurotypical female controls (mean age: 27.9±7.3 years; range: 18–52) were included and underwent MRI with DTI and neurobehavioural assessment at the Institute of Psychiatry, Psychology and Neuroscience, King's College London (males with ASC: 35; male controls: 33; females with ASC: 10; female controls: 21) or the Autism Research Centre, University of Cambridge (males with ASC: 26; male controls: 28; females with ASC: 27; female controls: 33) as part of the UK Medical Research Council Autism Imaging Multicentre Study (MRC AIMS).

Inclusion criteria for the ASC group included a diagnosis of autism according to the International Statistical Classification of Diseases, 10th Revision (ICD-10) research criteria. A childhood diagnosis was confirmed using the Autism Diagnostic Interview-Revised (ADI-R).^28^ These interviews on retrospective childhood behaviors with parents or carers confirmed all individuals with ASC exceeded cutoff scores within the domains of social interaction, communication, and repetitive and stereotypical behaviors. However, failure to reach cutoff was permitted by one point in any one of the domains. Current symptoms within the domains of impaired communication and reciprocal social interaction were measured using the Autism Diagnostic Observation Schedule (ADOS), module 4.^29^ The ADOS is an observational assessment of standardized activities, which allows an examiner to observe behaviors of interest in an ASC diagnosis. The occurrence of behaviors and interactions during the activities is rated, with higher scores representing behavior more typically associated with ASC. As all study participants were adults, these observations represent current ASC severity. The Wechsler Abbreviated Scale of Intelligence^30^ was used to assess overall intellectual ability. All individuals reached full-scale intelligence quotient (IQ) values >70 (details in Table 1). Adults with a history of head injury, genetic disorder associated with autism (for example, fragile X syndrome or tuberous sclerosis) or other neurological conditions that may affect brain function (for example, epilepsy) were excluded from the study. Further, exclusion criteria included drug abuse (for example, alcohol) and regular use of mood stabilizers, benzodiazepines or current antipsychotic medications.

In accordance with ethics approval by the National Research Ethics Committee, Suffolk, England, written informed consent was obtained from all participants.

### DTI acquisition protocol and analyses

MRI scans were performed using a 3-tesla GE magnet and an 8-channel receive-only radio frequency head coil (GE Medical Systems HDx, King's College London, UK and University of Cambridge, UK). Diffusion weighted images were acquired with a spin-echo pulse sequence together with echo-planar readout providing 2.4 mm^3^ isotropic resolution and whole head coverage. A double refocusing pulse was used to reduce eddy current induced artefacts. A set of 60 slices without slice gap was obtained with a field of view of 30.7 × 30.7cm^2^ and an acquisition matrix of 128 × 128. At each slice location 6 non-diffusion-weighted and 32 diffusion-weighted volumes with different non-collinear diffusion directions with a *b*-value of 1300 s mm^−2^ were acquired. Using a peripheral gating device placed on the participants' forefinger, the acquisition was cardiac gated with a repetition time (TR) equivalent to 20R-R intervals and an echo time (TE) of 104.5 ms. More details on the acquisition sequence are provided by Jones *et al.*^31^

Pre-processing and generation of fiber tract data were performed using ExploreDTI.^32^ This consisted of correction for head motion and eddy current induced geometric distortions of raw diffusion-weighted data;^33^ further details can be found in Catani *et al.*^22^ Subsequently, the diffusion tensor was estimated in each voxel using a nonlinear least square method^34^ and fractional anisotropy (FA), a measure giving information on the degree of directionality of the diffusion tensor, was determined in each voxel.

As the number of streamlines and the tract volume may vary substantially between participants, we used a region of interest approach within a recent DTI atlas^35, 36, 37^ (http://www.natbrainlab.com). We coregistered individual whole-brain FA volumes to the FMRIB58 template using nonlinear registration as implemented in the FSL software package^38^ (http://www.fmrib.ox.ac.uk/fsl). Bilaterally, we defined five specific brain regions in each hemisphere in the FMRIB58 space containing fiber tracts originating in the frontal lobe: the cingulum (the fiber bundle that runs around the corpus callosum with the cingulated gyrus), UF (the bundle of fibers connecting the medial and lateral orbitofrontal cortex with the anterior temporal lobe), IFOF (the long ventral bundle running from the orbitofrontal cortex to the ventral occipital lobe) and anterior and long segments of the AF (anterior: connecting the precentral, inferior frontal and middle frontal gyri, known as Broca's territory, to Geschwind's territory in the supramarginal gyrus; long: the fiber bundle between Broca's territory and Wernicke's territory in the superior and middle temporal lobe). We also identified two non-frontal fiber bundles, the inferior longitudinal fasciculus (ILF; connecting the anterior temporal lobe to the central occipital lobe) and posterior segments of the AF (linking Geschwind's and Wernicke's territories), in order to identify between-group differences in FA.^39^ The tracts analyzed were based on recent findings of frontospecific abnormalities in adult males with ASC, which were absent in the ILF and posterior segments of the AF.^22^

### Statistical analyses

Statistical testing was undertaken using SPSS 20.0 (IBM, Armonk, NY, USA) in which statistical significance was defined as *P*<0.05 (two-tailed) for all analyses.

Independent sample *t*-tests were used to calculate demographic differences between sexes. To compare tract-specific FA values between groups, multivariate analysis of covariance (MANCOVA) models were used. In these models tract mean FA values served as dependent variables, diagnostic group and sex as fixed factors, and scanning centre, age and FSIQ were added as covariates. We also tested whether there was an interaction effect over-and-above the main effects of sex and diagnosis separately (that is, the effect of an ASC diagnosis differs in strength and/or direction between sexes). Holm–Bonferroni correction was applied to account for multiple comparisons.

To exclude current symptom severity (that is, determined by the ADOS) as the driving factor for significant interactions, we compared FA between ASC individuals who did and did not reach ADOS cutoff for ‘autism spectrum' (that is, ADOS Total score of 7) scores using a MANCOVA for each sex. In addition, we calculated Bayes factors *post hoc*. These factors represent a weighted measure of the plausibility of the prior hypothesis that there was no difference between groups, versus the presence of a significant difference.^40^ They are particularly useful in the interpretation of null results, as they can distinguish between the two underlying causes of a null result (that is, a real absence of differences, versus insensitivity of the investigated data to provide a significant result). For computing Bayes factors, a freely available calculator was used (http://www.lifesci.sussex.ac.uk/home/Zoltan_Dienes/inference/Bayes.htm) which required the data summary (that is, mean difference between FA of those who did and did not reach ADOS cutoff scores, per sex and the standard error of this difference) and specification of the theory tested against the null hypothesis. For the latter, a uniform distribution of plausibilities of population effects was assumed, with a lower limit of 0 and upper limit defined as the maximum observed difference. Bayes factor thresholds of 0.33 and 3 were applied, where values below 0.33 suggest the data support the prior hypothesis of no difference between groups, values above 3 support the alternative hypothesis, and values in between suggest the data are insensitive to draw conclusions from Dienes *et al.*^40^ In addition, we determined ASC-specific sex differences with further adjustment for ADOS Total scores (that is, ADOS Total is the sum of the Social Interaction and Communication scores); together, this informed us the effect current ASC severity had on tract-specific mean FA values. To ensure our study had sufficient power to detect significant sex differences after ADOS adjustment, *post hoc* power analyses were performed using the G*Power software package.^41^

## Results

### Participant demographics

ASC groups were matched for age and severity of childhood autistic symptoms (Table 1). ADOS scores were significantly higher in ASC males (ADOS Total score males: 9.4±4.3; females: 6.8±6.0, *P*=0.016). Full scale IQ (FSIQ) did not differ between sexes in the ASC groups, but FSIQ scores of female controls were higher than those of females with an ASC diagnosis and both male diagnostic groups. Comparisons between verbal and performance IQ scores showed similar results. To adjust for the IQ differences, FSIQ was included as a covariate for all following analyses.

### Sex-specific effects and sex-by-diagnosis interaction effects

Comparison of tract mean FA values of male and female controls revealed comparable microstructural integrity levels in all frontal tracts. However, of the non-frontal tracts, the right ILF was shown to have significantly higher mean FA in females, FA=0.44004, than males, FA=0.43191 (*F*_(1,113)_=6.82, *P*=0.010).

**Figure 1:**
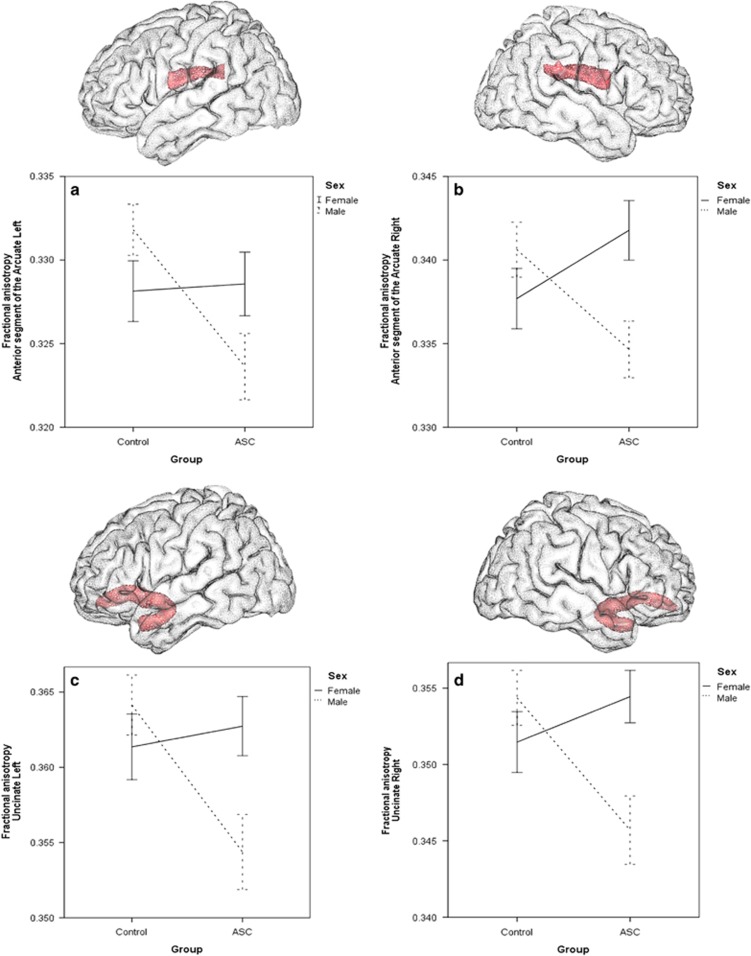
Visualizations of investigated tracts and mean fractional anisotropy graphs showing significant sex-by-diagnosis interaction effects. (**a**). Left anterior segment of the AF; (**b**) right segment of the AF; (**c**) left UF; (**d**) right UF. Bars indicate s.e. AF, arcuate fasciculus; ASC, autism spectrum condition; UF, uncinate fasciculus.

In males we found significant diagnostic effects of lower tract mean FA values in the ASC group compared to neurotypical controls in all frontal tracts except the long segment of the right AF (*F*_(1,120)=_0.60, *P*=0.444) and all investigated non-frontal tracts (Table 2). No significant diagnostic effects were found between the female ASC and control groups. Significant interaction effects were found for all frontal tracts (Figure 1) (suggesting that the diagnostic group effects in males are significantly different from the diagnostic group effects in females) except for the right long segment of the AF. The non-frontal tracts revealed no sex-by-diagnosis interactions (Table 2).

### Effects of ASC severity

To explore how current symptom severity influenced our results, we first completed analyses between those who scored above and below ADOS ‘autism spectrum' cutoff (that is, ADOS Total score of 7)^29^ within both males and females with a childhood autism diagnosis, as confirmed by the ADI-R.^28^ ADOS cutoff groups only differed on levels of current symptom severity; they were age and IQ matched. These analyses revealed no significant differences within either sex. To further explore this null result, Bayes factors were computed. These supported the findings of no difference (N.B. Bayes factors for the left UF and right IFOF in males, and left posterior segment of the AF in females, exceeded the set threshold of 0.33; Table 3).

We subsequently investigated whether correcting for ADOS scores altered the sex effect on tract differences within the ASC group. In this analysis we focussed on tracts with significant interaction values to minimize multiple comparisons effects. We found that all differences remained significant after this adjustment (Table 4). Given our sample size (*N*=57: total number of males and females for whom the ADOS total score was available) and number of groups (*k*=2: sex), a power analysis on the ADOS adjusted ASC-specific sex differences suggested that the observed power of sex differences varied between 0.53 and 0.95 at a specified alpha level of 0.05.

## Discussion

We report sex differences in frontal lobe connectivity in ASC. More specifically, we report frontal abnormalities in adult males with ASC that are absent in adult females with ASC. These results are consistent with previous volumetric and diffusion imaging findings^10, 11^ and provide further support to the *a priori* hypothesis that sex differences in the behavioral phenotype of ASC might be underpinned by differences in brain connectivity.

### Alternative explanations for sex differences in brain connectivity

The neuroanatomical differences found suggest intrinsic differences in WM organization of adult females with ASC compared to their male counterparts. In addition, a normative sex difference was found in the right ILF of control subjects, highlighting the presence of structural differences in brain connectivity independent of an ASC diagnosis. However, the sex differences in ASC were unique to connections originating from the frontal lobe. The frontal specificity of our finding is of potential importance because of the involvement of the frontal lobe in higher-order cognitive functioning affected in ASC, and the postulated ‘disconnection syndrome' underlying ASC during development.^20, 21, 22^ The neuroanatomical sex differences observed in the current study may partially account for the different behavioral phenotype of ASC females.^7^

It could also be argued that our findings are due to a skewed pattern of ASC symptom severity. It has been proposed, for example, that in order for women to reach the threshold for a clinical ASC diagnosis, they require the presence of more severe brain abnormalities as they are better able to compensate for, or mask their autistic disabilities than men.^3, 4, 5, 6^ This hypothesis is supported by some findings of greater structural brain abnormalities^42, 43^ and a greater genetic mutation load^44^ in females with ASC. To minimize this potential effect, we matched the male and female groups on the severity of their childhood ASC symptoms (that is, ADI-R scores^28^) as opposed to their current symptom severity (that is, ADOS scores^29^). A consequence of this approach was a sex bias with fewer women scoring above ADOS cutoff than men. To determine whether this difference accounted for our findings, we first carried out within-sex analyses based on scoring above or below ADOS cutoff. These analyses revealed the absence of mean FA differences in any of the tracts based on ADOS status in either sex. This suggests that current symptom severity does not modulate the FA values of frontal tracts. Further *post hoc* analyses also found that, after correction for the ADOS scores, the sex-by-diagnosis interactions remained significant. However, these analyses were underpowered for some tracts (for example, the left anterior AF segment, right cingulum and left UF) and larger studies are still needed to verify these findings.

Another issue to consider is the developmental nature of ASC. Although the observed variance in WM organization in our adult sample might represent an innate sex difference, it is also plausible that it is secondary to other experiential factors. For example, due to culturally defined sex differences, girls with ASC may receive more social interaction, and subsequently adopt more intrapersonal skills than boys.^45^ This may exert a protective effect on ASC etiology and/or a modulating effect on neurodevelopment in females.^46^ Equally, early diagnosis of ASC in males and under-detection of the condition in females may lead to differences in the pharmacological management of common co-morbidities (for example, depression, anxiety and attention deficit/hyperactivity disorder) during development. Differential exposure to medications could in turn influence critical periods of brain development, such as myelination and pruning.^47^ Finally, sex-specific physiological features, such as sex hormones (see below), may also affect sexual differentiation of the brain.^48^ Longitudinal studies of ASC are required to elucidate the sex-specific effects of these factors on lifespan development in individuals with ASC.

### Possible biological explanations: biological differences

ASC is a complex condition that involves multiple genetic variations. The biological basis of sex differences in frontal brain connectivity in ASC may additionally involve an interaction between sex hormones and sex chromosomes. It has been hypothesized, for example, that genes on the paternal X chromosome protect against social and communication impairments. This protective effect is absent in males due to their inheriting a single maternal X chromosome.^49^ It has also been postulated that differential peaks of testosterone during prenatal neurodevelopment may predispose to sex differences in vulnerability to autism.^50^ Fetal testosterone concentration has been reported to be positively associated with a number of autistic traits in neurotypical males and females.^51, 52^ Fetal testosterone also influences brain structures associated with language and communication in boys with ASC.^53^ Our findings therefore raise the question of whether (fetal) testosterone modulates the neurodevelopment of frontal connectivity in ASC. Modulation of frontotemporal functional connectivity by testosterone levels has already been reported in neurotypical individuals,^54^ but to date we are unaware of any studies on the putative effects of fetal testosterone on WM organization. In brief, the contribution of sexual differentiation mechanisms to sex-specific risks of developing ASC should be a key area for future studies.^2^

Future investigations should also include other regions of interest and WM connections beyond those analyzed in the present study. These could, for example, include the cerebellum^9, 16^ and temporoparietal junction^10, 17^ as both regions have previously been reported to exhibit sex differences in white and/or gray matter volume. Such studies may also benefit from the application of a 2 × 2 factorial design and TBSS. The main advantage of TBSS is that it is a fully automated, operator-independent approach that allows a ‘whole brain' analysis of global patterns of white matter integrity. It therefore has the potential to identify WM differences in brain regions not previously considered to be of importance and is resistant to operator-bias.

## Conclusion

We report sex differences in brain connectivity in ASC, with frontal abnormalities in adult males with ASC that are absent in adult females with ASC. These differences may explain some of the sex differences reported in the behavioral phenotype of ASC. Larger and longitudinal studies are required to replicate these findings and to explore differences in brain connectivity between other brain regions that could contribute to the sex differences seen in behavioral phenotypes.

## Figures and Tables

**Table 1:** Demographics of study cohort

	*ASC males (*n=*61) mean*±*s.d., [range]*	*Male controls (*n=*61) mean±s.d., [range]*	*ASC females (*n=*37) mean±s.d., [range]*	*Female controls (*n=*54) mean±s.d., [range]*	*Statistics* P*-value*^a^
Age, years	26.0±7.0, [18–41]	28.5±6.8, [18–45]	25.4±6.1, [18–37]	27.9±7.3, [18–52]	MC>FA (*P*=0.028)
FSIQ, WASI	115.3±12.6, [77–137]	110.9±11.8, [88–133]	113.7±15.0, [73–136]	120.0±7.9, [99–137]	MA<FC (*P*=0.016), MC<FC (*P*<0.001), FA<FC (*P*=0.024)
PIQ, WASI	115.2±13.8, [75–138]	111.3±13.1, [84–138]	109.1±15.8, [67–137]	116.0±9.1, [96–134]	MA>FA (*P*=0.048), MC<FC (*P*=0.027), FA<FC (*P*=0.020)
VIQ, WASI	112.3±12.6, [71–137]	108.3±13.0, [84–139]	115.3±15.7, [67–144]	119.1±9.1, [96–141]	MA<FC (*P*=0.001), MC<FA (*P*=0.019), MC<FC (*P*<0.001)
ADI-R Total^b^	36.7±9.2, [20–57]		33.5±9.0, [21–64]		NS
ADI-R Social^b^	18.1±5.4, [9–28]		16.3±4.6, [10–29]		NS
ADI-R Communication^b^	13.7±4.3, [8–24]		12.8±4.4, [7–25]		NS
ADI-R Repetitive Behavior^b^	4.9±2.2, [2–10]		4.4±1.9, [2–10]		NS
ADOS Total^c^	9.4±4.3, [1–21]		6.8±6.0, [0–22]		MA>FA (*P*=0.016)
ADOS Social Interaction^c^	6.1±3.0, [1–14]		4.7±3.8, [0–14]		MA>FA (*P*=0.042)
ADOS Communication^c^	1.2±1.2, [0–5]		2.2±2.3, [0–8]		MA>FA (*P*=0.008)

Abbreviations: ADI-R, Autism Diagnostic Interview-Revised; ADOS, Autism Diagnostic Observation Schedule; ASC, autism spectrum condition; FA, females with ASC diagnosis; FC, female controls; FSIQ, Full-scale IQ; IQ, intelligence quotient; MA, males with ASC diagnosis; MC, male controls; WASI, Wechsler Abbreviated Scale of Intelligence.

a *P*-values were not corrected for multiple comparisons. n.s, not significant (*P*>0.05). When Levene's Test for Equality of Variances showed significant non-equal variances, equal variance was not assumed.

b Information was available for all ASC participants. ADI-R Total is the sum of the Social interaction, Communication and Repetitive Behaviour scores for which respectively cutoff values of 10, 8 and 3 were used. Cutoff was not reached by 1 point for 2 male participants in the social interaction domain, 1 female participant in the communication domain and by 6 male and 6 female participants in the repetitive behavior domain.

c Information was available for 59 male ASC participants. ADOS Total is the sum of the Social Interaction and Communication scores for which cutoff values of 7, 4 and 2 are used, respectively; 43 male and 14 female individuals passed ADOS cutoff scores for ASC.

**Table 2:** Diagnosis effects and the sex-by-diagnosis interaction effect on fractional anisotropy values in frontal and non-frontal connectivity tracts

*Frontal tracts*	*Diagnostic effect in males*		*Diagnostic effect in females*		*Sex-by-diagnosis interaction*	
	*F*	P*-value*	*F*	P*-value*	*F*	P*-value*
Anterior segment AF left	12.75	0.001^a^	0.09	0.764	8.80	0.003^a^
Anterior segment AF right	8.90	0.003^a^	1.56	0.215	10.97	0.001^a^
Long segment AF left	7.21	0.008^a^	0.18	0.675	5.32	0.022^a^
Long segment AF right	0.60	0.444	0.32	0.573	1.22	0.272
Cingulum left	13.15	<0.001^a^	0.01	0.943	7.67	0.006^a^
Cingulum right	9.87	0.002^a^	0.01	0.937	5.66	0.018^a^
Uncinate left	14.13	<0.001^a^	0.30	0.588	9.86	0.002^a^
Uncinate right	12.06	0.001^a^	1.37	0.245	12.33	0.001^a^
IFOF left	11.80	0.001^a^	0.09	0.771	5.89	0.016^a^
IFOF right	10.75	0.001^a^	0.00	0.988	6.04	0.015^a^
*Non-frontal tracts*						
Posterior segment AF left	5.06	0.026^a^	0.00	0.975	2.73	0.100
Posterior segment AF right	4.30	0.040^a^	0.11	0.737	3.14	0.078
ILF left	8.92	0.003^a^	0.70	0.408	3.08	0.081
ILF right	6.02	0.016^a^	0.01	0.946	2.82	0.095

Abbreviations: AF, arcuate fasciculus; IFOF, inferior frontal occipital fasciculus; ILF, inferior longitudinal fasciculus; IQ, intelligence quotient.

Scanning centre, age and full scale IQ, were all included as covariates.

a *P*-values are significant at a level <0.05 after Holm-Bonferroni correction.

**Table 3:** Sex-specific differences between fractional anisotropy values in adults with an ASC diagnosis with and without severe current symptoms as measured using the ADOS total score

*Frontal tracts*	*Males (N=59) 16 ADOS− and 43 ADOS+*			*Females (N=36) 22 ADOS− and 14 ADOS+*		
	*F*	P*-value*	*Bayes factor*	*F*	P*-value*	*Bayes factor*
Anterior segment AF left	0.37	0.546	0.23	1.90	0.178	0.02
Anterior segment AF right	1.13	0.293	0.19	0.92	0.346	0.05
Long segment AF left	0.59	0.444	0.25	0.71	0.407	0.15
Long segment AF right	0.11	0.742	0.08	0.21	0.648	<0.01
Cingulum left	0.27	0.607	0.13	0.48	0.492	0.04
Cingulum right	0.03	0.870	0.01	0.12	0.734	0.02
Uncinate left	0.01	0.939	0.41^a^	3.48	0.072	0.01
Uncinate right	0.29	0.593	0.09	0.80	0.377	0.02
IFOF left	0.36	0.550	0.12	0.02	0.899	0.15
IFOF right	0.32	0.577	0.45^a^	0.09	0.761	0.09
*Non-frontal tracts*						
Posterior segment AF left	0.35	0.557	0.13	0.05	0.826	0.54^a^
Posterior segment AF right	0.06	0.802	0.19	0.43	0.515	0.03
ILF left	0.13	0.718	0.05	0.23	0.634	0.32
ILF right	0.18	0.671	0.21	0.93	0.342	0.05

Abbreviations: ADOS, Autism Diagnostic Observation Schedule; ADOS−, ASC participants not reaching ADOS cutoff score of 7; ADOS+, ASC participants reaching ADOS Total cutoff score of 7; AF, arcuate fasciculus; ASC, autism spectrum conditions; IFOF, inferior frontal occipital fasciculus; ILF, inferior longitudinal fasciculus; IQ, intelligence quotient.

Scanning centre, age, and full scale IQ, were all included as covariates.

a Bayes factors (>0.33) indicate data sensitivity was insufficient to draw conclusions from.

**Table 4:** ASC-specific sex differences in fractional anisotropy values of frontal tracts corrected for current symptom severity as measured using the ADOS total score

*Frontal tracts*	*Sex difference ADOS corrected*		*Observed power*
	*F*	P*-value*	
Anterior segment AF left	4.02	0.048^a^	0.53
Anterior segment AF right	8.83	0.004^a^	0.86
Long segment AF left	8.80	0.004^a^	0.86
Cingulum left	6.80	0.011^a^	0.76
Cingulum right	4.64	0.034^a^	0.60
Uncinate left	4.70	0.033^a^	0.60
Uncinate right	7.01	0.010^a^	0.77
IFOF left	8.11	0.005^a^	0.83
IFOF right	12.29	0.001^a^	0.95

Abbreviations: ADOS, Autism Diagnostic Observation Schedule; AF, arcuate fasciculus; ASC, autism spectrum conditions; IFOF, Inferior Frontal Occipital Fasciculus; IQ, intelligence quotient.

Scanning centre, age and full scale IQ, were all included as covariates.

a *P*-values are significant at a level <0.05.
